# Endogenous ceramide phosphoethanolamine modulates circadian rhythm via neural–glial coupling in *Drosophila*

**DOI:** 10.1093/nsr/nwac148

**Published:** 2022-07-27

**Authors:** Xiupeng Chen, Jie Li, Zhongbao Gao, Yang Yang, Wenqing Kuang, Yue Dong, Gek Huey Chua, Xiahe Huang, Binhua Jiang, He Tian, Yingchun Wang, Xun Huang, Yan Li, Sin Man Lam, Guanghou Shui

**Affiliations:** State Key Laboratory of Molecular Developmental Biology, Institute of Genetics and Developmental Biology, Chinese Academy of Sciences, Beijing100101, China; University of Chinese Academy of Sciences, Beijing 100049, China; State Key Laboratory of Molecular Developmental Biology, Institute of Genetics and Developmental Biology, Chinese Academy of Sciences, Beijing100101, China; University of Chinese Academy of Sciences, Beijing 100049, China; University of Chinese Academy of Sciences, Beijing 100049, China; State Key Laboratory of Brain and Cognitive Science, Institute of Biophysics, Chinese Academy of Sciences, Beijing 100101, China; University of Chinese Academy of Sciences, Beijing 100049, China; State Key Laboratory of Brain and Cognitive Science, Institute of Biophysics, Chinese Academy of Sciences, Beijing 100101, China; State Key Laboratory of Molecular Developmental Biology, Institute of Genetics and Developmental Biology, Chinese Academy of Sciences, Beijing100101, China; University of Chinese Academy of Sciences, Beijing 100049, China; State Key Laboratory of Molecular Developmental Biology, Institute of Genetics and Developmental Biology, Chinese Academy of Sciences, Beijing100101, China; University of Chinese Academy of Sciences, Beijing 100049, China; LipidALL Technologies Company Limited, Changzhou213022, China; State Key Laboratory of Molecular Developmental Biology, Institute of Genetics and Developmental Biology, Chinese Academy of Sciences, Beijing100101, China; LipidALL Technologies Company Limited, Changzhou213022, China; State Key Laboratory of Molecular Developmental Biology, Institute of Genetics and Developmental Biology, Chinese Academy of Sciences, Beijing100101, China; State Key Laboratory of Molecular Developmental Biology, Institute of Genetics and Developmental Biology, Chinese Academy of Sciences, Beijing100101, China; University of Chinese Academy of Sciences, Beijing 100049, China; State Key Laboratory of Molecular Developmental Biology, Institute of Genetics and Developmental Biology, Chinese Academy of Sciences, Beijing100101, China; University of Chinese Academy of Sciences, Beijing 100049, China; University of Chinese Academy of Sciences, Beijing 100049, China; State Key Laboratory of Brain and Cognitive Science, Institute of Biophysics, Chinese Academy of Sciences, Beijing 100101, China; State Key Laboratory of Molecular Developmental Biology, Institute of Genetics and Developmental Biology, Chinese Academy of Sciences, Beijing100101, China; LipidALL Technologies Company Limited, Changzhou213022, China; State Key Laboratory of Molecular Developmental Biology, Institute of Genetics and Developmental Biology, Chinese Academy of Sciences, Beijing100101, China; University of Chinese Academy of Sciences, Beijing 100049, China

**Keywords:** ceramide phosphoethanolamine, sphingomyelin, circadian, longevity, glutamate homeostasis

## Abstract

While endogenous lipids are known to exhibit rhythmic oscillations, less is known about how specific lipids modulate circadian behavior. Through a series of loss-of-function and gain-of-function experiments on ceramide phosphoethanolamine (CPE) synthase of *Drosophila*, we demonstrated that pan-glial-specific deficiency in membrane CPE, the structural analog of mammalian sphingomyelin (SM), leads to arrhythmic locomotor behavior and shortens lifespan, while the reverse is true for increasing CPE. Comparative proteomics uncovered dysregulated synaptic glutamate utilization and transport in CPE-deficient flies. An extensive genetic screen was conducted to verify the role of differentially expressed proteins in circadian regulation. Arrhythmic locomotion under *cpes^1^* mutant background was rescued only by restoring endogenous CPE or SM through expressing their respective synthases. Our results underscore the essential role of CPE in maintaining synaptic glutamate homeostasis and modulating circadian behavior in *Drosophila*. The findings suggest that region-specific elevations of functional membrane lipids can benefit circadian regulation.

## INTRODUCTION

The circadian clock entrains rhythmic patterns in behavioral and physiological processes to temporally coordinate systemic metabolism with the rising and setting of the sun [1]. Importantly, it controls daily oscillations in metabolic flux, feeding–fasting cycle and sleep–wake activity, and plays a critical role in aging and various other metabolic diseases [2,3]. Cellular metabolism is intimately connected with the circadian clock [4,5] and a myriad of endogenous metabolites was shown to exhibit rhythmic oscillations in a circadian fashion [6–8]. In our previous investigation on human subjects, sphingomyelins (SMs) exhibited strong rhythmicity on a per-individual basis [8]. In a separate study, the rhythmic cycling of SMs in the suprachiasmatic nucleus of mice was found to be exclusively influenced by a high-fat diet [9]. While the circadian clock entrains metabolic processes that can result in cyclic patterns of metabolites [10], oscillating patterns of endogenous metabolites may also underlie the circadian modulation of cellular behavior and metabolism [4,11,12]. Preceding works showed that cyclic ADP-ribose fine-tunes the circadian cycle of cytosolic calcium release and contributes to the expression of circadian clock genes in plant and animal cells [13,14], while flavin adenine dinucleotide modulates the clock protein CRYPTOCHROME (CRY) and regulates the circadian oscillation of metabolic genes in mammals [15].

*Drosophila* is a model organism for investigating circadian rhythm, as several clock components are conserved in flies [16]. *Drosophila* is, by far, the most advanced model to investigate the intricate biochemical interplay underlying circadian regulation and a large number of the characterized clock genes were identified via genetic screening using rhythms in spontaneous locomotor activity as a circadian output [17]. Eclosion (adult emergence from pupal case) and adult locomotor activity denote two behaviors widely employed for assessment of circadian rhythmicity in *Drosophila* [18]. The brain of *Drosophila* contains ∼150 neurons that intertwine to form a master pacemaker network, which governs locomotor activity through sensing zeitgeber inputs and entrains clock neuron oscillations via neural signals [19–21]. Morning (M) and evening (E) cells generate M and E activity bouts during the light–dark cycles [22,23]. The clock genes in these neurons, including *period* (*per*), *timeless* (*tim*), *Clock* (*Clk*), *Par domain protein 1ϵ* (*pdp1ϵ*), *vrille* (*vri*) and *cycle* (*cyc*), constitute positive and negative feedback loops through transcriptional/translational activation and inhibition to govern rhythmic output, and form the molecular mechanisms underlying *Drosophila* circadian oscillators [24–26].

SM, or its analog in *Drosophila*, i.e. CPE, forms the major sphingolipid component of the plasma membranes of animal cells [27] and is ubiquitously distributed in all tissues with enriched levels in the central nervous system [28,29]. While the mammalian system mainly comprises SM with only a trace amount of CPE, the *Drosophila* lipidome has largely replaced SM with CPE with respect to both structural and signaling functions [30,31]. CPES (encoded by *CG4585*) and *Drosophila* SM synthase-related protein (dSMSr) largely govern CPE biosynthesis in flies, in contrast to SM biosynthesis in the mammalian system predominantly mediated by SM synthase 1 and 2 (SMS1 and SMS2) [32–34]. Common to both mammalian and *Drosophila* systems, acid and neutral sphingomyelinases (aSMase and nSMase) mediate the conversion of CPE (or SM) into ceramides based on the pH of the microenvironment [35,36]. A previous study showed that *Drosophila cpes* mutants exhibited photosensitive epilepsy [31]. The mechanistic connection between SM and circadian regulation in *Drosophila*, however, is unclear to date. Furthermore, whether SM (or its analog CPE) could underlie the modulation of circadian patterns and rhythmic behavior has largely remained unknown.

In the current study, we sought to elucidate the role of CPE in modulating circadian regulation using *Drosophila* as a model via systematically manipulating the expression of genes implicated in CPE biosynthesis and metabolism. Corroborating our preceding observation in human subjects, we showed that the CPE level in astrocyte-like glia (ALG) is essential for the maintenance of circadian rhythm in *Drosophila*. Tissue-specific gain- and loss-of-function assays further confirmed that the aberrant circadian phenotype is ALG-dependent. Comparative proteomics uncovered the potential role of glutamate excitotoxicity mediated by the Na^+^/K^+^-dependent excitatory amino acid transporter EAAT1 in leading to arrhythmic locomotion in *cpes*-RNAi flies, which also exhibited compromised lifespan. Our findings demonstrate that CPE production in ALG modulates circadian rhythm in locomotor activity and lifespan in *Drosophila*, possibly via CPE-enriched membrane microdomains in this subpopulation of glial cells essential for proper maintenance of the extracellular synaptic space.

## RESULTS

### Null-allele mutants of genes mediating CPE biosynthesis display perturbed circadian rhythm

**Figure 1.**
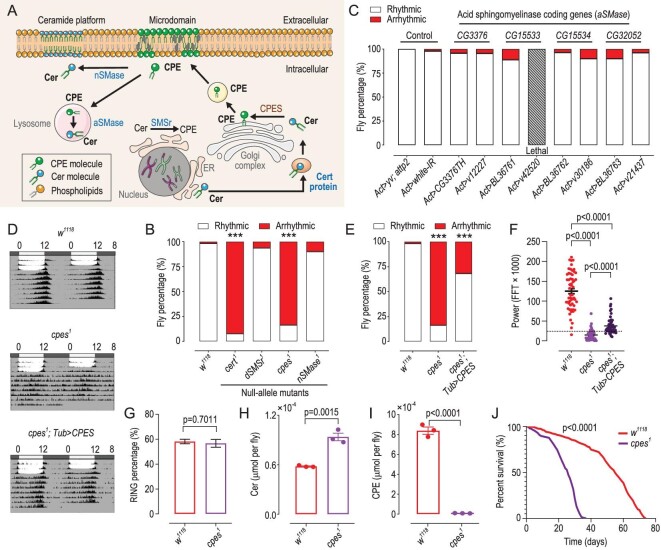
*cpes* regulates spontaneous locomotor activity in *Drosophila*. (A) Schematic model illustrating CPE metabolism in *Drosophila.* Ceramide synthesized on the ER is transferred by Cert protein to the Golgi complex, where ceramide is converted to CPE mediated by CPES. A trace mount of CPE was produced by dSMSr on ER. Plasma membrane CPE can be hydrolysed into ceramide via the action of nSMase, or aSMase under an acidic microenvironment (e.g. lysosomal compartment). Microdomain: cholesterol and sphingomyelin enriched liquid ordered phase on plasma membrane, which forms a docking site for many proteins. Ceramide platform: a special microdomain enriched with Cer and with a gel-like structure. Cer, ceramide; CPE, ceramide phosphoethanolamine; Cert, ceramide transfer protein; nSMase, neutral sphingomyelinase; aSMase, acidic sphingomyelinase; dSMSr, *Drosophila* SM synthase-related protein; CPES, ceramide phosphoethanolamine synthase. (B) Percentage of rhythmic and arrhythmic flies in *w^1118^* (*n* = 50), *cert^1^* (*n* = 39), *dSMSr^1^* (*n* = 32), *cpes^1^* (*n* = 43) and *nSMase^1^* (*n* = 31) mutants. *P*-values from Fisher's exact test are indicated, where ***P < 0.001. *cert*, ceramide transfer protein; *nSMase*, neutral sphingomyelinase encoding gene; *dSMSr, Drosophila* SM synthase-related protein encoding gene; *cpes*, ceramide phosphoethanolamine synthase encoding gene. (C) Percentage of rhythmic and arrhythmic flies in *Actin-Gal4*-derived knock-down of genes encoding aSMase, where *Act > yv; attp2* (*n* = 45) and *Act > white-IR* (*n* = 47) served as control groups. *Ac* (*n* = 23), *Act > v12227* (*n* = 22), *Act > BL36761* (*n* = 28), *Act > v42520* (lethal), *Act > BL36762* (*n* = 28), *Act > v30186* (*n* = 30), *Act > BL36763* (*n* = 30), *Act > v21437* (*n* = 26). *Act* refers to *Actin-Gal4*. BL denotes Bloomington *Drosophila* stock number, while v denotes Vienna *Drosophila* Resource Center stock number, TH refers to Tsinghua Fly Center. (D)–(E) Averaged double-plot actograms (D) and percentage of rhythmic and arrhythmic flies in *w^1118^* (*n* = 50), *cpes^1^* mutants (*n* = 52) and *cpes^1^; Tub > CPES* rescue flies (*n* = 44) (E). *w^1118^*, wild-type; *Tub, Tub-Gal4; cpes^1^*, ceramide phosphoethanolamine mutants. *P*-values of mutant and rescue flies compared to *w^1118^* using the Fisher's exact test are illustrated, where ***P < 0.001. (F) Circadian powers calculated by Fast Fourier Transform (FFT) of *Drosophila* locomotion patterns, with flies showing a FFT × 1000 value > 25 counted as rhythmic. Fly number is as same as (E). *P*-values from one-way ANOVA with Tukey's post-hoc test are indicated. (G) RING assay analysis of geotaxis in *w^1118^* and *cpes^1^.* Data presented in each group are averaged results from five independent tubes each containing 30 flies. Flies in each tube were tested for four or five consecutive rounds. Data are presented as mean ± SEM; Student's *t*-test. RING, Rapid Iterative Negative Geotaxis. (H) and (I) HPLC-MS/MS quantitation of the levels (μmol per fly) of ceramide (H) and CPE (I) in the whole-body samples from *w^1118^* and *cpes^1^* mutants. *n* = 3 independent samples each containing two flies for each group. Data were analysed using Student's *t*-test. Cer, ceramide; CPE, ceramide phosphoethanolamine. (J) Kaplan–Meier survival analysis indicates that lifespan was significantly reduced (*P* < 0.0001) in *cpes^1^* mutants relative to *w^1118^*flies. A total of ∼240 flies were included at the start of the survival analysis for each group, where the survival curve represents the percentage of surviving flies in each group at each time point. The number of surviving flies was recorded at 1- or 2-day intervals. *P*-value from the Log-rank (Mantel-Cox) test is indicated.

We first sought to confirm whether CPE affects the maintenance of the circadian clock by employing the CRISPR/Cas9 technology to construct various mutants of genes implicated in the biosynthesis and metabolism of CPE in *Drosophila* (Fig. 1A), monitoring fly locomotor rhythm with the pySolo system as a paradigm of circadian output [37–39]. The stability of the locomotor rhythm measurement system was first verified by using circadian rhythm mutants *per^01^, Clk^Jrk^* and *Pdf^01^* as positive controls (Supplementary Fig. S1A-C). Results from null-allele mutants showed that compared with *w^1118^*, there was no significant difference in the percentages of arrhythmic flies in *dSMSr^1^* (*CG32380*) and *nSMase^1^* (*CG12034*) mutants, while *cpes^1^* (*CG4585*) and *cert^1^* (*CG7207*) mutants showed arrhythmic phenotypes (Fig. 1B). To date, four acidic sphingomyelinases (aSMase) have been reported in *Drosophila*, and *CG3376* encodes the main aSMase located in the lysosome. All *CG3376* null-alleles mutants are, however, homozygous lethal. Next, we used the UAS/Gal4 system to knock-down *CG3376* and other potential aSMase encoding genes (*CG15533, CG15534, CG32052*) with ubiquitously expressed *Actin5C-Gal4*. In ubiquitous knock-down flies, *CG3376* RNAi (BL 36760) and *CG15533* RNAi (v42520) are lethal and thus we obtained a new Transgenic RNAi Project (TRiP) line named *CG3376TH* instead. All these strains, however, did not exhibit appreciable changes in circadian rhythms (Fig. 1C).

Notably, we found that male *cpes* mutants displayed a perturbed circadian phenotype defined by complete arrhythmic locomotor activity (Fig. 1D–F and Supplementary Fig. S1D), while their locomotor response (i.e. geotaxis) as determined by the Rapid Iterative Negative Geotaxis (RING) assay is comparable to that of wild-type (Fig. 1G). These observations support that *cpes* deficiency disrupts circadian regulation in *Drosophila*.

We next assessed the levels of endogenous CPEs and their precursor lipids ceramide in *cpes^1^* mutants using targeted lipidomics. Compared with *w^1118^*, total CPE was reduced by ∼98%, coupled with an appreciable increase in ceramide precursors (Fig. 1H and I). We also noted that ∼76% of *cpes^1^* mutants exhibited dorsal closure defects in the abdomen (Supplementary Fig. S1E and F) and all male *cpes^1^* homozygous mutants are sterile. Interestingly, the lifespan of *cpes^1^* mutants was significantly shortened relative to *w^1118^* flies (Fig. 1J). Thus, we subsequently focused on elucidating how genetically manipulating the expression of *cpes*, which is responsible for the bulk of CPE production in *Drosophila* [30,31], regulates circadian rhythm in flies.

### *cpes* downregulation but not overexpression disrupts spontaneous locomotor rhythm

**Figure 2.**
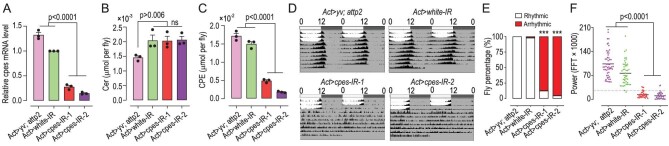
*cpes* ubiquitous knock-down alters spontaneous locomotor rhythm. (A) RT-qPCR result of relative levels of *cpes* expression in *Actin-Gal4*-derived knock-downs. *Act > yv; attp2* and *Act > white-IR* denote control groups; *Act > cpes-IR-1* and *Act > cpes-IR-2* are two independent RNAi strains of the *cpes* gene. *n* = 3 independent RNA samples for each genotype, where each RNA sample contained RNA extract from 30 flies. *P*-values from one-way ANOVA followed by post-hoc Tukey's test are indicated. Error bars represent mean ± SEM. (B) and (C) Endogenous levels of ceramide (B) and CPE (C) quantitated by lipidomics in *Actin-Gal4*-driven knock-downs. *Act > yv; attp2, Act > white-IR* denote control groups. *n* = 3 independent samples each containing two flies for each group. *P*-values from one-way ANOVA followed by post-hoc Tukey's test are indicated. Error bars represent mean ± SEM. Cer, ceramide; CPE, ceramide phosphoethanolamine. ns denotes *P* > 0.05. (D)–(F) Disrupted spontaneous locomotor rhythm in *cpes*-RNAi strains relative to controls. Averaged double-plot actograms in the two RNAi strains *Act > cpes-IR-1* (*n* = 41), *Act > cpes-IR-1* (*n* = 42) and control groups in *Act > yv; attp2* (*n* = 53), *Act > white-IR* (*n* = 47) *cpes* knock-downs compared with *Act > white-IR* (D), percentage of rhythmic and arrhythmic flies (E), circadian powers calculated using Fast Fourier Transform (FFT) of *Drosophila* locomotion patterns, with flies having a FFT × 1000 value > 25 counted as rhythmic (F). *P*-values from Fisher's exact test are indicated in (E) and one-way ANOVA with post-hoc Tukey's test are indicated in (F). ***P < 0.001.

To eliminate the influence of genetic background on the circadian rhythm of *cpes^1^*, we constructed two *cpes*-RNAi (IR) strains using the TRiP method targeting different mRNA regions [40,41]. Quantitative real-time PCR (RT-qPCR) was used to assess knock-down efficiency in ubiquitous knock-downs. The levels of *cpes* mRNA in the two strains of *Actin > cpes-RNAi* (*Act > cpes-IR*) flies raised at 25^o^C were reduced substantially compared with control genotypes (Fig. 2A). Lipidomics analysis revealed no significant changes in ceramide levels compared with the *Act > white-IR* control group (Fig. 2B), while CPE levels were reduced by >68% (Fig. 2C). Consistently with the phenotype observed in *cpes* null-allele mutants, >80% of the knock-downs exhibit dorsal closure defects (Supplementary Fig. S2A and B). Examining lipid changes confined to the brain regions, the levels of CPE in the two knock-down strains were >90% reduced, while no significant difference in ceramide levels was observed (Supplementary Fig. S2C–E). Thus, the *cpes-IR* strains are ideal for investigating the effect of specifically reducing endogenous CPE levels on circadian regulation in *Drosophila*. In dark–dark (DD) cycles, both *Act > cpes-IR* lines displayed disrupted oscillations and very low circadian power, i.e. below the defined threshold (FFTX1000 < 25), whereas the two control groups (*Act > yv; attp2* and *Act > white-IR*) exhibited normal circadian-entrained spontaneous locomotor rhythm (Fig. 2D and F). Comparable to *cpes^1^* mutants, the percentages of arrhythmic flies in *cpes* ubiquitous knock-downs were close to 90% (Fig. 2E).

We next examined whether *cpes* gain-of-function could disrupt circadian regulation. *UAS-CPES* was driven by *Actin-Gal4* in flies reared at 25^o^C. As expected, relative mRNA levels of *cpes* and endogenous CPEs were appreciably increased in the overexpression line compared to control groups (Supplementary Fig. S3A and B). In addition, increasing endogenous CPEs did not result in appreciable changes in locomotor activity, circadian period nor strength compared to control groups at Day 5 after eclosion (Supplementary Fig. S3C–E). On another note, we observed an increase in the lifespan of *Act > CPES* flies, which was extended for ∼20 days compared to controls (Supplementary Fig. S3F). The increase in the lifespan of the *cpes* overexpression line was in contrast to that of *Act > cpes-IR* flies, which exhibited significantly shortened lifespan (∼35 days) compared to controls (Supplementary Fig. S3G). The foregoing results showed that keeping the endogenous level of CPE above a physiological threshold is essential for maintaining spontaneous locomotor rhythm in *Drosophila*. In addition, decreasing the endogenous level of CPE in flies has a negative impact on lifespan, and vice versa.

### Glial-cell-specific downregulation of *cpes* leads to disrupted circadian clock

**Figure 3.**
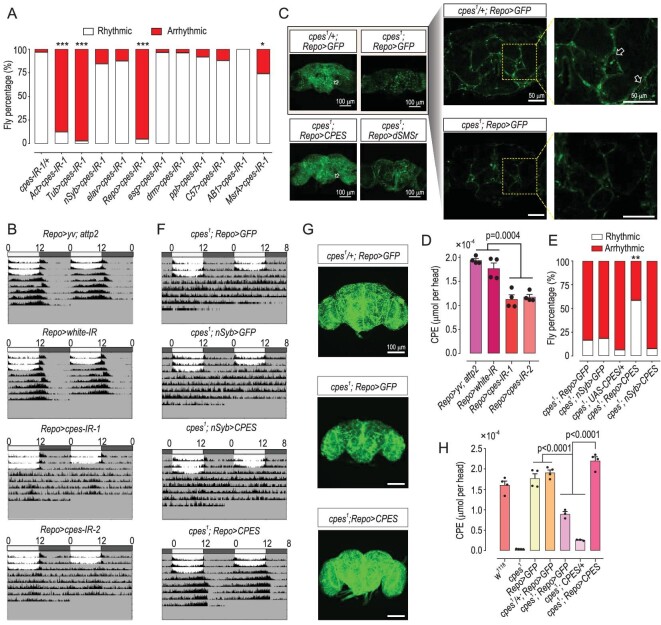
Disrupted spontaneous locomotor rhythm in *cpes* mutant is mediated by pan-glial cells. (A) Percentage of rhythmic and arrhythmic flies in different region-specific RNAi strains including neuron: *nSyb-Gal4* (*n* = 26) and *elav-Gal4* (*n* = 24), pan-glial: *Repo-Gal4* (*n* = 42), gut: *esg-Gal4* (*n* = 29) and *drm-Gal4* (*n* = 26), body fat: *ppl-Gal4* (*n* = 24), muscles: *C57-Gal4* (*n* = 25), salivary gland: *AB1-Gal4* (*n* = 27), epidermis: *MrsA-Gal4* (*n* = 27), relative to control group *cpes-IR-1/+* (*n* = 31) and ubiquitous knock-downs: *Actin-Gal4* (*n* = 40) and *Tubulin-Gal4* (*n* = 35). *P*-values from Fisher's exact test are indicated, where ***P < 0.001, *P < 0.05. (B) Averaged double-plot actograms in pan-glial-specific *cpes*-RNAi *Repo > cpes-IR-1*(*n* = 18) and *Repo > cpes-IR-2* (*n* = 18) relative to control groups *Repo > yv; attp2* (*n* = 18) and *Repo > white-IR* (*n* = 18). (C) Confocal images of pan-glia in control (*cpes^1^/+; Repo > GFP*) and mutant (*cpes^1^; Repo > GFP*) compared to rescue lines (*cpes^1^; Repo > CPES*) and (*cpes^1^; Repo > dSMSr*). Composite images presented are representative images from two independent experiments. Scale bar = 100 μm. Visible glia meshworks are indicated with white open arrows in control and *CPES* rescue flies. Single optical sections of neuropil regions in controls (*cpes^1^/+; Repo > GFP*) and mutants (*cpes^1^; Repo > GFP*) are enlarged in the upper panel on the right. Glia processes emanating from cell bodies are indicated with white open arrows in controls, which were visibly weaker in intensity in mutants. Scale bar = 50 μm. *Repo-Gal4* linked with *UAS-mCD8:: gfp*. (D) HPLC-MS/MS quantitation of the levels (μmol per head) of CPE in the brain regions of pan-glial-specific-*cpes* knock-downs relative to control groups. *n* = 4 independent biological replicates each containing five fly heads. *P*-values from one-way ANOVA with post-hoc Tukey's test are indicated. Error bars represent mean ± SEM. CPE, ceramide phosphoethanolamine. (E) Percentage of rhythmic and arrhythmic flies in glial-cell-specific and neuron-specific rescue strains. *cpes^1^; Repo > GFP* (*n* = 30), *cpes^1^; nSyb > GFP* (*n* = 27) and *cpes^1^; CPES/+* (*n* = 31) represents control group and *cpes^1^; Repo > CPES* (*n* = 29) and *cpes^1^; nSyb > CPES* (*n* = 26) denote glial-cell specific and neuron-specific rescue flies, respectively. *P*-values from Fisher's exact test between *cpes^1^; Repo > GFP* and *cpes^1^; Repo > CPES* were indicated, where **P < 0.01. (F) Averaged double-plot actograms in tissue-specific rescues under *cpes^1^* mutant background. Glia-specific *cpes^1^; Repo > CPES* (*n* = 18) and neuron-specific *cpes^1^; nSyb > CPES* (*n* = 18) rescue strains compared to the controls*, cpes^1^; Repo > GFP* (*n* = 10) and *cpes^1^; nSyb > GFP* (*n* = 9). Only expression of *cpes* in glial cells restored rhythmic locomotor activity. (G) Confocal microscopy images of glia morphology in *cpes^1^/+; Repo > GFP* (wild-type control) and *cpes^1^; Repo > GFP* (mutants) and *cpes^1^; Repo > CPES* (glial-cell rescue). Green color illustrates *Repo-gal4* linked with *UAS-mCD8:: gfp. Repo* is short for *Repo-mCD8:: gfp.* Images presented are representative images from three independent experiments. Scale bar = 100 μm. (H) CPE levels analysed using HPLC-MS/MS in different genotypes, *n* = 3 or 4 biological replicates per group, where each replicate comprises five fly heads. Error bars represent mean ± SEM, *P*-values from one-way ANOVA with Tukey's post-hoc test.

To determine the tissue-specificity of the CPE-mediated regulation of the circadian rhythm, we used Gal4 lines driving clock neuron-specific knock-down of *cpes* (*per-Gal4, tim-Gal4, Clk4.1M-Gal4, pdf-Gal4, pdfr-Gal4, cry-Gal4, R81H11-Gal4*). Results showed that there were no significant differences in the spontaneous locomotor rhythm in flies with clock neuron-specific reductions in CPE compared to *cpes-IR-1/+* (Supplementary Fig. S4A). While ubiquitous knock-downs of *cpes* driven by both *Actin-Gal4* and *Tubulin-Gal4* resulted in altered locomotor rhythm, neuron-specific knock-downs with *nSyb-Gal4* and *elav-Gal4* had no significant effect on circadian regulation. Interestingly, we found that knock-down of *cpes* specifically in pan-glial cells driven by *Repo-Gal4* resulted in arrhythmicity comparable to ubiquitous knock-downs. Furthermore, in most other cell-specific knock-downs investigated, including the gut (*esg-Gal4, drm-Gal4*), fat body (*ppl-Gal4*), muscles (*C57-Gal4*) and the salivary gland (*AB1-Gal4*), no significant perturbation in circadian rhythm was observed compared to control flies (*cpes-IR-1*/+), except for a slight perturbation (∼25%) observed in the epidermis (*MrsA-Gal4*). These observations justify that glial-cell-specific expression of *cpes* is of utmost importance to entraining the circadian clock in *Drosophila* (Fig. 3A).

To eliminate off-target and false-positive effects, we replicated the results by including another *cpes*-RNAi strain (*cpes-IR-2*) and compared the changes in spontaneous locomotor activity with control flies of different genetic backgrounds (Fig. 3B and Supplementary Fig. S4B). The circadian powers of both *RNAi* lines fell below the defined threshold [Fast Fourier Transform (FFT) × 1000 = 25] (Supplementary Fig. S4C). We also observed that the density of the glial mesh (white, open arrows) in both *cpes^1^* mutants (*cpes^1^; Repo > GFP*) and pan-glia-specific *cpes* knock-downs (Fig. 3C and Supplementary Fig. 4G) were visibly reduced compared to controls, suggesting that CPE may be important in the maintenance of glial cells. As expected, CPE decreased by ∼36% in the brain regions of *cpes* knock-downs, while no significant changes were observed for ceramide (Fig. 3D and Supplementary Fig. 4D). In agreement with the phenotypes observed in *cpes* ubiquitous knock-downs and *cpes^1^* mutants, glial-cell-specific *cpes* knock-downs exhibited significantly shortened lifespan (Supplementary Fig. S4E).

To validate that circadian disruption was caused by *cpes* deficiency specifically in glia, we expressed *UAS-CPES* with *Repo-Gal4* and *nSyb-Gal4* in pan-glia and pan-neurons under *cpes^1^* background, respectively. While glial-cell-specific rescue partially restored rhythmic locomotor activity, no restoration was noted for *nSyb > CPES* (Fig. 3E and F, and Supplementary Fig. 4F), again demonstrating that the expression of *cpes* in glia plays an essential role in circadian entrainment. Besides, *cpes^1^; Repo > CPES* restored the density of the glial meshwork (white, open arrow) in neuropil regions, while expression of *dSMSr* did not (Fig. 3C and G). As expected, the endogenous CPE levels in glial rescue flies were restored to comparable levels as wild-type flies, and significantly higher than that in the *cpes^1^* mutant (Fig. 3H). No obvious changes were observed for ceramide (Supplementary Fig. S4I). Lipidomics results showed that all individual CPE species were similarly restored in the glial-cell-specific rescue of *cpes^1^* mutants independently of acyl-chain lengths and sphingoid base identities (Supplementary Fig. S4H). Overexpression of *cpes* in glial cells from the zygote stage can extend lifespan by almost 30 days (Supplementary Fig. S4J) and conditional induction of *cpes* overexpression in the glia of aged flies (at 35 days after eclosion) by controlling housing temperature could also prolong lifespan (Supplementary Fig. S4K). We further monitored locomotor rhythm in aged flies and found that circadian power was enhanced in aged flies overexpressing *cpes* in their glial cells compared to age-matched controls (Supplementary Fig. S4L and M), indicating that increasing the level of CPE in glial cells helps to sustain circadian regulation in aged flies.

Taken together, these results revealed that a diminished level of CPE in glial cells is responsible for arrhythmic locomotor activity in both *cpes^1^* mutants and knock-downs, and suggested that glial abnormality resulting from CPE deficiency may lead to aberrant circadian behavior, with implications also on lifespan and aging.

### ALG denotes the key subtype of pan-glial cells underlying arrhythmic circadian clock

**Figure 4.**
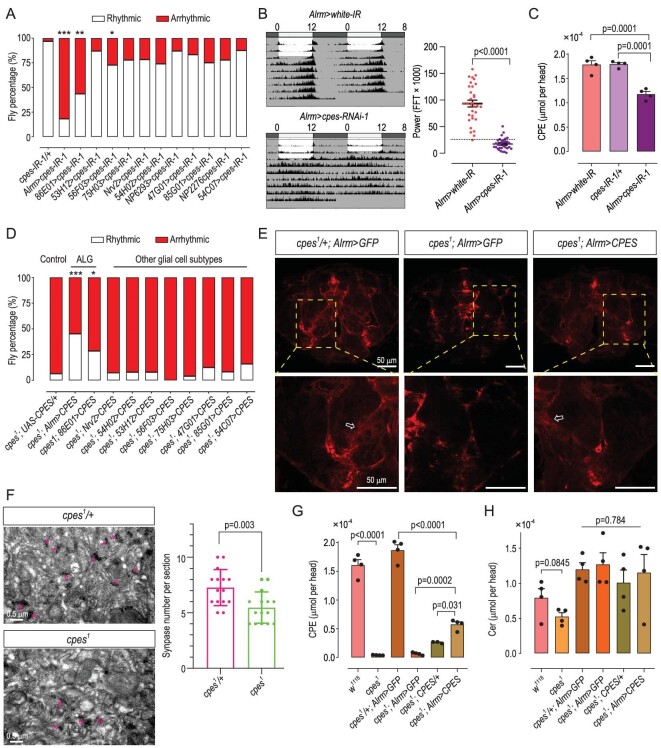
CPE reductions in astrocyte-like glial cells (ALG) underlie circadian disruption in *cpes* mutants. (A) Percentage of rhythmic and arrhythmic flies in *cpes* knock-downs of different glia subtypes. Astrocyte-like glial *Alrm-Gal4* (*n* = 44) and *R86E01-Gal4* (*n* = 55), ensheathing glial *R53H12-Gal4* (*n* = 23), *R56F03-Gal4* (*n* = 22), *R75H03-Gal4* (*n* = 27), cortex glial cells *Nrv2-Gal4* (*n* = 25), *R54H02-Gal4* (*n* = 23), perineurial glial cells *NP6293-Gal4* (*n* = 24), *R47G01-Gal4* (*n* = 24) and *R85G01-Gal4* (*n* = 25), subperineurial glial cells *NP2276-Gal4* (*n* = 28), *R54C07-Gal4* (*n* = 24). *P*-values from Fisher's exact test are indicated, *P < 0.05, **P < 0.01, ***P < 0.001. (B) Averaged double-plot actograms of ALG-specific *cpes* knock-downs compared to controls. *Alrm > white-IR* (*n* = 9), *Alrm > cpes-RNAi-1* (*n* = 9). Circadian power in ALG-specific *cpes* knock-down *Alrm > cpes-IR-1* (*n* = 32) compared to control *white-IR* (*n* = 32). *P*-value from Student's *t*-test is indicated. (C) Brain CPE levels in ALG-specific knock-downs compared to controls. Data (μmoles) were normalized to head number, where *n* = 4 biological replicates per group; each replicate comprises five heads. Error bars represent mean ± SEM; *P*-values from one-way ANOVA with Tukey's post-hoc test. (D) Percentage of rhythmic and arrhythmic flies of CPES rescues in different glial-cell subtypes including *cpes^1^; UAS/+* (*n* = 31), *cpes^1^; Alrm > CPES* (*n* = 31), *cpes^1^;86E01 > CPES* (*n* = 35), *cpes^1^; Nrv2 > CPES* (*n* = 27), *cpes^1^;54H02 > CPES* (*n* = 25), *cpes^1^;53H12 > CPES* (*n* = 25), *cpes^1^;56F03 > CPES* (*n* = 25), *cpes^1^;75H03 > CPES* (*n* = 24), *cpes^1^;47G01 > CPES* (*n* = 24), *cpes^1^;85G01 > CPES* (*n* = 24), *cpes^1^;54C07 > CPES* (*n* = 25). *P*-values from Fisher's exact test are indicated, *P < 0.05, ***P < 0.001. (E) Confocal microscopy images of ALG in control (*cpes^1^/+; Alrm > GFP*), mutant (*cpes^1^; Alrm > GFP*) and rescue flies (*cpes^1^; Alrm > CPES*). *Alrm*: *Alrm-mCD8:: ChRFP* (*Alrm-Gal4* is linked with *UAS- mCD8:: ChRFP*). Higher-magnification images of neuropil regions (boxed) are presented in the lower panel. Scale bar = 50 μm. Images presented are representative images from three independent experiments. (F) Synapses imaged using transmission electron microscopy (TEM) in the brain sections of *cpes^1^/+*control (*n* = 3) and mutant *cpes^1^*(*n* = 3). Images are representative of three independent experiments. Synapses were identified by ultrastructural criteria and indicated with magenta-pink open arrows. The number of synapses per section was counted for five sections from each independent experiment, making a total of 15 sections for *cpes^1^/+* control and mutant *cpes^1^*, respectively. Number of synapses per section was compared between *cpes^1^/+* control and mutant *cpes^1^* using Student's *t*-test. (G) and (H) Levels of CPE (G) and ceramide (H) quantified using HPLC-MS/MS, where *n* = 4 biological replicates each comprising five heads; error bars represent mean ± SEM, *P*-values from one-way ANOVA with Tukey's post-hoc test.

The pan-glial cells of *Drosophila* can be divided into five major subtypes [42,43]. To determine the specific glial subtype responsible for arrhythmic locomotor activity observed in glial-specific *cpes* knock-downs, we deployed a series of Gal4 drivers to specifically knock down *cpes* in the five major glia subtypes, including ALG (*Alrm-Gal4* and *R86E01-Gal4*), ensheathing glial (*R53H12-Gal4, R56F03-Gal4, R75H03-Gal4*), cortex glial cells (*Nrv2-Gal4, R54H02-Gal4*), perineurial glial cells (*NP6293-Gal4, R47G01-Gal4* and *R85G01-Gal4*), as well as subperineurial glial cells (*NP2276-Gal4, R54C07-Gal4*) [44,45]. Our results showed that only ALG-specific knock-down of *cpes* led to appreciable and maximum perturbations (i.e. both *Alrm-Gal4* and *R86E01-Gal4*) in spontaneous locomotor rhythm (Fig. 4A–C). To rule out a potential effect from genetic background, we validated our findings using another *cpes* knock-down strain (*Alrm > cpes-IR-2*) compared with control flies (*Alrm > white-IR*) (Supplementary Fig. S5A–D). Lipidomics revealed that CPE in the brain regions of both *Alrm > cpes-IR-1* (Fig. 4C) and *Alrm > cpes-IR-2* (Supplementary Fig. S5E) were decreased by >30% relative to control groups, supporting that the ALG-specific reductions in CPE underlie perturbations in spontaneous locomotor rhythm. To confirm the role of ALG in the CPE-dependent regulation of circadian rhythm, we specifically expressed *UAS-CPES* in different glial subtypes under a *cpes^1^* mutant background. We found that only *CPES* expression in ALG (*cpes^1^; Alrm > CPES* and *cpes^1^;86E01 > CPES*), but not other glial subtypes, partially but significantly rescued the arrhythmic locomotion (Fig. 4D). We next examined the general morphology of ALG by expressing *UAS-mCD8-ChRFP* driven by *Alrm-Gal4* to specifically label the ALG cell membrane (Fig. 4E). As our labeling strategy labels all ALG cells, the morphology of individual cells cannot be distinguished, but it confers a general overview of the density of glial processes (sparse or dense), as indicated by fluorescence intensity [44]. As revealed by higher-magnification images of the neuropil regions on the lower panel, the dense glia mesh (white open arrow) in *cpes^1^/+ Alrm > GFP* was almost absent in *cpes^1^; Alrm > GFP*, and partially restored (white open arrow) in *cpes^1^; Alrm > CPES* (Fig. 4E). Given the primary role of ALG in modulating brain synapses [44] and preceding findings that *Drosophila* ALG is pro-synaptogenic, i.e. ALG ablation reduces synapse numbers [46], we then examined and counted the number of synapses in the brain of control *cpes^1^/+* and mutant *cpes^1^* flies using transmission electron microscopy (TEM) (Fig. 4F). The numbers of synapses in the brain were significantly reduced in mutant flies (Fig. 4F). Thus, confocal imaging in combination with TEM suggests a reduced number of ALG cells in *cpes^1^* mutants relative to controls. In line with the imaging and behavioral results aforementioned, the brain CPE levels of ALG-specific rescue flies increased significantly relative to different *cpes* mutants, albeit not reaching the levels in controls (Fig. 4G). No appreciable changes were noted for ceramides (Fig. 4H). Thus, ALG-specific deficiency in CPE leads to reductions in the density of ALG structural processes and a reduced number of ALG cells, coupled with perturbed circadian rhythm.

### *cpes* knock-down in glia alters molecular oscillations of clock genes

Transcriptional oscillations of clock genes including *per, tim, clk, Pdp1ϵ, vri* and *cry* in pacemaker neurons govern physiological and behavioral rhythms [16,26]. Given the validated disruptions in the spontaneous locomotor rhythm in *cpes* knock-downs, we then measured the changes in clock gene expressions in glia-specific *cpes* knock-downs relative to control flies comprising mixed *Repo > white-IR* and *Repo > yv; attp2* at a 1:1 ratio. We collected *Repo > cpes-IR* (*Repo > cpes-IR-1* and *Repo > cpes-IR-2*) at 4-h intervals under 12-h light/12-h dark (LD) and constant darkness (DD) conditions, which began at Zeitgeber time zero (ZT0) or Clock time zero (CT0, light on at 8:00 a.m.). RT-qPCR was used to quantify mRNA levels of clock genes at different time points. Our results showed that the oscillations of all clock genes in *Repo > cpes-IR* were appreciably lower than that in the control group under the LD cycle (Supplementary Fig. S6A–F). Under DD, most clock genes in the knock-down flies displayed similar trends in periodicity despite weaker oscillations compared to the control group, with the exception of *Clk*, which exhibited a virtually opposite phase in knock-down relative to control flies (Supplementary Fig. S6G–L). In addition, the mRNA levels of *Pigment-dispersing factor* (*Pdf*) in knock-down flies exhibited an ∼4.5-fold increase compared to control flies under both LD and DD (Supplementary Fig. S5F). In all, RT-qPCR results showed that the molecular oscillations in clock pacemakers were altered in glial-specific *cpes* knock-downs and that the arrhythmic locomotor activity phenotype in *cpes* knock-down flies was mediated by changes in transcriptional oscillations of clock genes. The transcriptional levels and catalytic activity of CPES, however, did not display appreciable rhythmicity (Supplementary Fig. S6M and N).

### Circadian disruption in *cpes^1^* mutants depends on CPES catalytic activity

**Figure 5.**
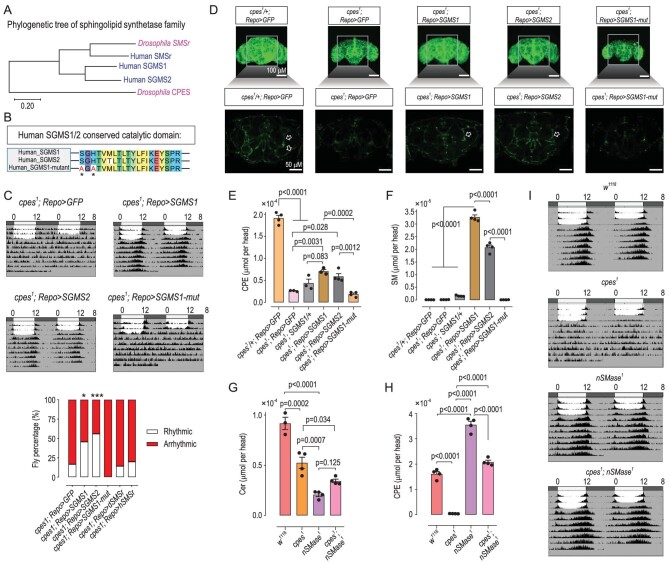
Levels of endogenous CPEs, not ceramides, underlie circadian perturbation in *cpes^1^* mutants. (A) Phylogenetic tree of SM and CPE synthetases. Protein sequences of dSMSr, CPES and human SGMS1, SGMS2 and hSMSr were comparatively analysed using MEGA-X. (B) Conserved catalytic domain in human SGMS1 and SGMS2 proteins and amino acid mutations in SGMS1-mutant (S283A, H285A) are asterisked. (C) Averaged double-plot actograms in human SGMS1/2 *cpes^1^; Repo > SGMS1* (*n* = 9), *cpes^1^; Repo > SGMS2* (*n* = 9) and catalytic dysfunctional *cpes^1^; Repo > SGMS1-mut* (*n* = 9) rescue flies under *cpes^1^* mutant background *cpes^1^; Repo > GFP* (*n* = 18). Percentage of rhythmic and arrhythmic flies in different genotypes. Fly numbers: *cpes^1^; Repo > GFP* (*n* = 30), *cpes^1^; Repo > SGMS1* (*n* = 35), *cpes^1^; Repo > SGMS2* (*n* = 50), *cpes^1^; Repo > SGMS1-mut* (*n* = 24), *cpes^1^; Repo > dSMSr* (*n* = 28), *cpes^1^; Repo > hSMSr* (*n* = 30). *P*-values from Fisher's exact test are indicated, *P < 0.05, ***P < 0.001. (D) Upper panel: composite confocal images of pan-glia in control flies (*cpes^1^/+; Repo > GFP*), mutants (*cpes^1^; Repo > GFP*) and rescue flies of SGMS. Green color denotes *Repo-gal4* linked with *UAS-mCD8:: gfp. Repo* is short for *Repo-mCD8:: gfp*. Images presented are representative images from three independent experiments. Scale bar = 100 μm. Lower panel: single optical sections of neuropil regions; glia processes emanating from cell bodies are indicated with white open arrows. Scale bar = 50 μm. (E) and (F) Brain levels of CPE (E) and SM (F) quantified by HPLC-MS/MS in different genotypes, where *n* = 3 or 4 biological replicates per group; each replicate contains five fly heads. Error bars represent mean ± SEM. *P*-values from one-way ANOVA with Tukey's post-hoc test. SM, sphingomyelin. (G) and (H) Brain levels of ceramide (G) and CPE (H) quantified by HPLC-MS/MS, where *n* = 3 or 4 biological replicates per group; each replicate contains five fly heads. Error bars represent mean ± SEM. *P*-values from one-way ANOVA with Tukey's post-hoc test. (I) Averaged double-plot actograms of control *w^1118^* (*n* = 18), mutants *cpes^1^* (*n* = 18), *nSMase^1^* (*n* = 18) and rescue flies *cpes^1^; nSMase^1^* (*n* = 18). *nSMase^1^* mutation could reverse arrhythmic locomotor activity under *cpes^1^* mutant background, with *cpes^1^; nSMase^1^* flies displaying virtually normal locomotor rhythm.

SGMS1 and SGMS2 are bona fide sphingomyelin synthases in mammals, which contain four highly conserved sequence motifs (D1–D4), and point mutation in the structural motif HHD can completely abrogate their catalytic activity [47]. SGMS1 produces the bulk of cellular SM in mammalian trans-Golgi lumen, while SGMS2 is located in the plasma membrane and is responsible for the synthesis of SM and CPE [48]. Phylogenetic tree of SM and CPE synthases showed that SGMS1/2 and CPES are closely related (Fig. 5A). To investigate whether arrhythmic locomotor activity in *cpes* mutant and knock-down flies is dependent on the catalytic activity of CPES or via protein physical interactions, we introduced a transgene with a point mutation in the conserved catalytic domain of human sphingomyelin synthase1, i.e. *UAS-SGMS1-mut.* The *UAS-SGMS1-mut* transgenic flies possess an abolished D3 (C-G-D-X3-S-G-H-T) motif, with S-G-H mutated to A-G-A, where histidine H constitutes part of the structural HHD motif critical for the catalytic function of SGMS1 (Fig. 5B). Arrhythmic locomotor activity was partially but significantly rescued via expression of catalytically functional *SGMS1* and *SGMS2* in glia, but not for *cpes^1^; Repo > SGMS1-mut* (Fig. 5C). In a similar light, the intensity of green fluorescence from glial tissues in the neuropil regions was restored in flies expressing *SGMS1* and *SGMS2*, but not for catalytically dysfunctional *SGMS1-mut* (Fig. 5D). These results indicate that SGMS1/2 could functionally replace CPES in *Drosophila* and the catalytic activity of CPES is critical in the regulation of circadian rhythm in flies. Similarly to *dSMSr*, expression of the human *SMSr* (*hSMSr*), however, did not rescue spontaneous locomotor rhythm (Supplementary Fig. S7A and B). This may be explained in part by considering that SMSr only contributes a minimal portion of the bulk CPE production in *Drosophila* [32,49] or that CPE produced by SMSr is ER-localized and could not be transported to plasma membrane under normal physiological circumstances. Importantly, lipidomics showed that the levels of CPE increased by ∼50% in both SGMS1 and SGMS2 rescue fly brains relative to different *cpes* mutant flies (Fig. 5E). Interestingly, these two rescue lines exhibited elevated SM levels compared to other genotypes (Fig. 5F), indicating that SGMS1 and SGMS2 can catalyse the biosynthesis of both CPE and SM in *Drosophila*, and that SM may functionally replace CPE in *Drosophila* (Supplementary Fig. S7C and D). In addition, we also observed that flies containing *UAS-SGMS1* without the *Repo-Gal4* driver synthesized a small amount of SM and possessed a higher level of CPE under a *cpes^1^* mutant background, which implied a leakage expression of the UAS element in the absence of GAL4 (Fig. 5E and F). Thus, these results strongly indicated that arrhythmic locomotor activity and compromised glial phenotype in *cpes^1^* mutants depend on the catalytic activity of CPES.

### Plasma membrane depletion of CPE, but not ceramides, underlies arrhythmic locomotor rhythm

To further delineate the potential contribution of CPE versus ceramides to arrhythmic locomotor activity, we first measured their endogenous contents in *cpes^1^* brains. We found that both ceramides and CPE were significantly decreased in *cpes^1^* compared with *w^1118^* flies (Fig. 5G and H). We also noted that ceramide changes in *cpes^1^* brains displayed opposite trends compared to whole-body (i.e. increase) (Figs 1G and 2B). As structural analogs of SM, CPE may exhibit similar cellular distributions in *Drosophila* and share comparable biophysical attributes, such as the inherent biophysical property of SM to coalesce into microdomains in lipid bilayers. To further explore whether membrane depletion of CPE underlies arrhythmic locomotor activity in *cpes^1^* mutants, we generated *nSMase^1^* null-allele mutants via CRISPR/Cas9. We found that, similarly to *cpes^1^* mutant, ceramides in the brain region were decreased compared to *w^1118^*, whereas CPEs in *nSMase^1^* were increased by ∼120% relative to *w^1118^* (Fig. 5G and H). Notably, *nSMase^1^* flies display normal locomotor rhythm (Fig. 5I), which indicates that in the absence of diminished CPE levels, ceramide reduction per se does not lead to arrhythmic locomotor activity. We then crossed *cpes^1^* with *nSMase^1^* flies to investigate genetic interactions and found that the level of brain ceramides in the double mutant was partially restored but significantly lower than that of *w^1118^* (Fig. 5G). Notably, CPE in the brain was restored to a comparable level to that of *w^1118^* (Fig. 5G and H) and the introduction of the *nSMase^1^* completely rescued arrhythmic locomotor activity in the *cpes^1^* mutant (Fig. 5I and Supplementary Fig. 7E–G). Importantly, ceramides in *cpes^1^; nSMase^1^* were still lower than in *nSMase^1^* (Fig. 5G), which further supports that ceramide deficiency was not the main driver behind disrupted locomotor rhythm. A majority of the nSMase activity in the mammalian liver resides in the plasma membrane [50]. In line with these observations, we showed that expressing SGMS2, localized in the plasma membrane [31,51], could also rescue arrhythmic locomotor activity and aberrant glial phenotype under a *cpes^1^* mutant background (Fig. 5C and D). These results demonstrate that plasma membrane depletion of CPE, but not ceramides, underlies arrhythmic locomotor activity in glial-specific *cpes^1^* knock-downs.

### Membrane CPE depletion governing arrhythmic locomotor activity takes place prior to eclosion

**Figure 6.**
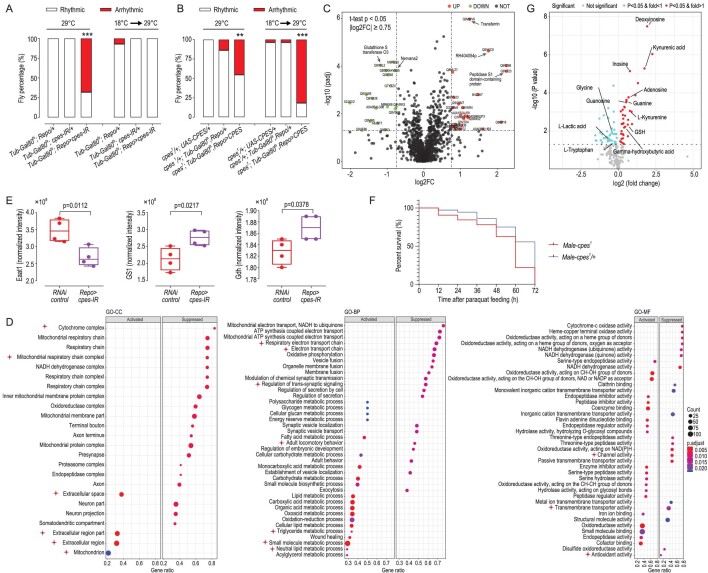
Comparative proteomics analysis in glia-specific *cpes* knock-downs revealed perturbed synaptic glutamate homeostasis. (A) Percentage of rhythmic and arrhythmic flies in conditional *cpes* knock-downs lines. *Tub-Gal80^ts^* was used to suppress *Repo-gal4* expression at 18^o^C. Knock-down flies raised at 29^o^C throughout development *Tub-Gal80^ts^; Repo > cpes-IR* (*n* = 25) were arrhythmic compared to controls *Tub-Gal80^ts^; Repo/+* (*n* = 25), *Tub-Gal80^ts^; cpes-IR/+* (*n* = 26) raised under the same conditions. Knock-down flies *Tub-Gal80^ts^; Repo > cpes-IR* (*n* = 30) first raised at 18^o^C then shifted to 29^o^C post-eclosion displayed normal rhythmic activity compared to controls *Tub-Gal80^ts^; Repo/+* (*n* = 28) and *Tub-Gal80^ts^; cpes-IR/+* (*n* = 27) raised under the same conditions. *P*-values from Fisher's exact test are indicated. *Tub-Gal80^ts^; Repo > cpes-IR* was compared to each control genotype independently, where ***P < 0.001. (B) Percentage of rhythmic and arrhythmic flies in *cpes* rescue lines. *UAS-CPES* expressed from the zygote stage could rescue arrhythmic patterns under *cpes^1^* mutant background, but not that expressed after eclosion at 29^o^C for 10 days. Rescue flies *cpes^1^; Tub-Gal80^ts^, Repo > CPES* (*n* = 31) raised at 29^o^C throughout development displayed partially rescued arrhythmic locomotion compared to controls *cpes^1^/+; UAS-CPES/+* (*n* = 27) and *cpes^1^/+; Tub-Gal80^ts^, Repo/+* (*n* = 29), whereas rescue flies *cpes1; Tub-Gal80^ts^, Repo > CPES* (*n = 33*) first raised at 18^o^C then shifted to 29^o^C post-eclosion displayed no rescue in arrhythmic locomotion compared to controls *cpes1/+; UAS-CPES/+* (*n = 28), cpes1/+; Tub-Gal80^ts^, Repo/+* (*n = 28). P*-value from Fisher's exact test is indicated, where **P < 0.01, ***P < 0.001. (C) Volcano plot illustrates differentially expressed proteins (DEPs), defined by |log_2_FC| ≥ 0.75 and *P* < 0.05, in glial-specific *cpes* pan-glia knock-downs compared to controls; *n* = 4 biological replicates per group and each replicate comprises five heads. Male flies 5–7 days after eclosion were acclimated for 3 days in an environment with 12-h light/12-h dark conditions, and were collected at 6:00 p.m. for proteomic analysis. *Repo > cpes-IR* denotes *Repo > cpes-IR-1* and *Repo > cpes-IR-2* mixed at a 1:1 ratio compared to RNAi control flies comprising *Repo > yv; attp2* and *Repo > white-IR* mixed at a 1:1 ratio. FC, fold-change in *Repo > cpes-IR* relative to RNAi control. (D) Enrichment plots illustrate top dysregulated pathways from cellular compartment (CC), biophysical processes (BP) and molecular functions (MF) categories curated in the Gene Ontology (GO) database. Gene ratio denotes the number of differentially expressed genes (proteins) relative to the total number of genes under the specific term. Pathways discussed in the text are asterisked to facilitate quick visualization. (E) Box plots illustrate comparisons of protein levels of EAAT1, GS1 and Gdh between glial-specific *cpes* knock-downs *Repo > cpes-IR* (*Repo > cpes-IR-1* and *Repo > cpes-IR-2* mixed at an equal ratio) and RNAi controls (*Repo > yv; attp2* and *Repo > white-IR* mixed at an equal ratio). *n* = 4 biological replicates per group and each replicate comprises five heads. In all box plots, the median is indicated by the horizontal line and the first and third quartiles are represented by the box edges. The lower and upper whiskers extend from the hinges to the smallest and largest values, respectively, with individual samples indicated as dots. *P*-values from Student's *t*-test are indicated. EAAT1, excitatory amino acid transporter 1; GS1, glutamine synthase 1; Gdh, glutamate dehydrogenase. (F) Paraquat resistance assay indicated elevated oxidative stress in *cpes^1^* mutants relative to controls. Male adult flies were exposed to a filter paper soaked with 20 mM paraquat (P814066, Macklin) in 5% sucrose solution. Survivorship of flies was recorded at every 12 h for a total of 72 h. *P*-value was from the Log-rank (Mantel-Cox) test. *n* = 39 flies for *cpes^1^/+* and *n* = 33 flies for *cpes^1^*. Male flies 5–7 days after eclosion were used for the assay. (G) Volcano plot illustrates differential metabolites as defined by *P* < 0.05 from Welch's *t*-test in *repo > cpes-RNAi* compared to controls *repo > yv; attp2*. Metabolites increased in *repo > cpes-RNAi* are indicated by red dots, while those reduced compared to controls are represented by blue dots. *n* = 4 biological replicates per group and each replicate comprises 25 heads. Male flies 5–7 days after eclosion were acclimated for 3 days in an environment with 12-h light/12-h dark conditions, and were collected at 6:00 p.m. for metabolomics analysis.

We next sought to discriminate whether the arrhythmic locomotor activity resulting from membrane CPE deficiency was mediated as an acute response or attributed to developmental events in glial cells by using temporal and regional gene-expression targeting methods to conditionally express *Repo-Gal4* under the control of *Tub-Gal80^ts^.* Gal80^ts^ could inhibit GAL4 function at 18^o^C but release the blockade at 29^o^C [52]. We showed that when flies were raised at 29^o^C from the zygote stage to eclosion, the percentage of arrhythmic flies in *Tub-Gal80^ts^; Repo > cpes-IR* (mixture of *cpes-IR-1* and *cpes-IR-2* flies at a 1:1 ratio) was higher than two control groups. When flies were only shifted to 29^o^C after eclosion and reared for 10 days, however, no appreciable disruption of spontaneous locomotor activity was seen in *Tub-Gal80^ts^; Repo > cpes-IR* compared to control groups (Fig. 6A and Supplementary Fig. 8A). These observations imply that CPE plays a crucial role in glia morphogenesis across *Drosophila* development or that CPE-mediated membrane aberrations governing the arrhythmic locomotor activity occur prior to eclosion. Indeed, when we expressed *UAS-CPES* under the control of *Tub-Gal80^ts^* in a *cpes^1^* mutant, arrhythmic locomotor activity was rescued only if these flies were grown at 29^o^C from the zygote stage, but not first at 18^o^C during development then shifted to 29^o^C post-eclosion (Fig. 6B and Supplementary Fig. 8B). It was not effective to restore CPE in the mature glial system to rescue arrhythmic locomotor activity and plasma membrane CPE depletion underlying arrhythmic locomotor activity was attributed to molecular events prior to eclosion rather than post-development. On another note, while CPE deficiency affects the development of glial cells, no gross morphological changes in neuronal populations were observed in *cpes* knock-downs and mutants (Supplementary Fig. S8C).

### Proteomics and metabolomics revealed altered synaptic glutamate transport and expenditure underlying arrhythmic locomotor activity in *cpes* knock-downs

We adopted an unbiased approach based on proteomics to elucidate key molecular drivers underlying arrhythmic locomotor activity in glial-specific *cpes* knock-downs relative to control flies. Differentially expressed proteins (DEPs) were investigated based on *t*-test *P* < 0.05 and a |log_2_FC| ≥ 0.75. The top significantly altered proteins with annotated functions in the UniProt database were labeled on the volcano plot (Fig. 6C). The Na^+^/K^+^ ATPase ion pump Nervana 2 (Nrv2) and glutathione S transferase O3 (GstO3) were amongst the top downregulated proteins in glia-specific *cpes* knock-downs, while transferrin and proteins possessing endopeptidase activity, such as RH404054p (SP99) and Peptidase S1 domain-containing protein (SP170), were upregulated. We then ranked the proteins based on test statistics and deployed gene set enrichment analysis (GSEA) to look for pathway perturbations, using gene sets originating from the Gene Ontology (GO) database downloaded from the Molecular Signature Database (MSigDB v7.3). GSEA GO-CC (cellular compartment) analysis revealed that the mitochondrion and extracellular region/space were amongst the top perturbed compartments. Several components of the electron transport chain critical for oxidative phosphorylation were downregulated in *cpes* knock-downs, including cytochrome complex and mitochondrial respiratory chain complex I (Fig. 6D). Indeed, GSEA GO-BP (biological process) pathway analysis supported that oxidative phosphorylation was suppressed in *cpes* knock-downs and the mitochondrial respiratory electron transport chain was negatively affected. Importantly, GSEA GO-BP analysis supported our preceding observations that adult locomotory behavior was suppressed in *cpes* knock-downs. A closer look into the top dysregulated proteins under this pathway uncovered galactose-1 phosphate uridylyltransferase (Galt), Tyrosine 3-monooxygenase (ple) and Na^+^/K^+^-dependent glutamate transporter EAAT1 (O77062) as key mediators of perturbed locomotor activity (Fig. 6E and Supplementary Fig. 8D), which were reduced in *cpes* knock-downs. EAAT1 represents the only high-affinity glutamate transporter in *Drosophila* (Rival *et al.*, 2006). We also noticed that a small molecule metabolic process was the top dysregulated pathway from GO-BP analysis. Amongst the top deregulated proteins under this pathway (ranked by ascending *P*-values) were several enzymes that expend glutamate, which were increased in *cpes-IR* mutants, including glutamine synthase 1 (GS1), glutamate dehydrogenase (Gdh) and Delta-1-pyrroline-5-carboxylate synthase (P5CS) that converts glutamate to glutamyl 5-phosphate (Fig. 6E and Supplementary Fig. 8D). GO-MF (molecular function) analysis revealed that metal-ion transmembrane transporter activity was suppressed in *cpes* knock-downs (Fig. 6D) in agreement with the observed reduction in Nrv2 based on *P*-value and fold-changes. In line with the reduction in glutathione transferase activity, GO-MF showed that antioxidant activity was abated in *cpes* knock-downs. To verify this, we examined the resistance of flies to oxidative damage on exposure to paraquat and found that the survival of *cpes^1^* mutants was significantly compromised compared to controls *cpes^1^/*+, indicating a lower capacity to cope with oxidative stress with *cpes* loss of function (Fig. 6F).

Thus, the brain of *cpes* knock-down flies appeared to experience elevated oxidative stress, compromised mitochondrial electron transport chain function and elevated protein breakdown via the action of endopeptidases. Mitochondria stress and redox imbalance denote characteristic features of glutamate excitotoxicity [53]. GO-BP also revealed that trans-synaptic signaling and synaptic vesicle transport, as well as the regulation of membrane potential, were downregulated in *cpes* knock-downs (Supplementary Dataset 1). Proteomics data therefore suggest that the activity of glial-localized glutamate transporter EAAT1, which couples glutamate uptake and clearance from the synaptic cleft to the action of the Na^+^/K^+^ ATPase pump (Nrv2) [54,55], may underlie the orchestration of circadian activity rhythms in *Drosophila* via modulating the extracellular synaptic milieu. We then performed untargeted metabolomics in the brains of *repo > cpes-RNAi* relative to control *repo > yv; attp2* flies to elucidate changes in the levels of various neurotransmitters and their related polar metabolites. Differential metabolites were illustrated in a volcano plot (Fig. 6G). Relative to control flies, numerous amino acids and purine metabolites were increased in the brains of glia-specific *cpes* knock-downs, while most acylcarnitines were reduced. In particular, lactate was reduced by 35% (*P* = 0.02), which, together with the reductions in various acylcarnitines, were concordant with our proteomics analysis that suggest compromised mitochondria function in the brain of glia-specific *cpes* knock-downs. Substantial increases in various purines, such as adenosines, were noted in glia-specific *cpes* knock-downs. A previous study had reported an extracellular release of purines triggered by calcium influx in response to activation of glutamate receptors [56], which may serve to modulate the presynaptic release of glutamate. Adenosines act as neuromodulators by binding to presynaptic A1 inhibitory receptors that inhibit the release of a majority of neurotransmitters including glutamate [57–59]. Moreover, drastic increases in kynurenic acid by 235% (*P* = 4.9 × 10^–7^) and L-kynurenine by 70% (*P* = 2.3 × 10^–4^) were observed in glia-specific *cpes* knock-downs, accompanied by significant reductions in their precursor substrate L-tryptophan (*P* = 0.02). Kynurenic acid acts as a glutamate antagonist with preferential action at the glycine residue of *N*-methyl-D-aspartate receptors (NMDARs) [60] and increases in kynurenic acids may henceforth denote compensatory responses to protect against glutamate-induced excitotoxic injury in *cpes* knock-downs. While we observed no significant changes in glutamate at the whole-brain level, the level of glutathione (GSH) was elevated by 54% (*P* = 0.003) in glia-specific *cpes* knock-downs. Biosynthesis of GSH utilizes glutamate and glycine as precursors, and the latter was also reduced by 26.5% (*P* = 0.005) in knock-down flies. Cerebral GSH is an antioxidant that protects neurons against free-radical attacks under an excitotoxic microenvironment [61]. Finally, while the levels of gamma-aminobutyric acid (GABA) were not significantly altered, the levels of gamma-hydroxybutyric acid (GHB) were appreciably increased by 36.7% (*P* = 0.003) in glia-specific *cpes* knock-downs. As a putative neurotransmitter derived from GABA, GHB inhibits both NMDAR- and AMPAR-mediated excitatory postsynaptic potentials in neurons [62].

### Extensive genetic screening identifies aberrant transynaptic signaling and clearance of glutamate behind perturbed circadian rhythm

**Figure 7.**
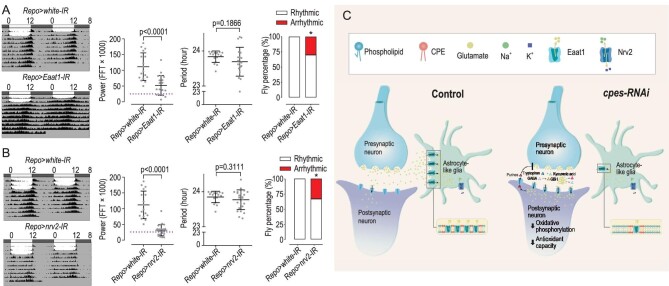
Nrv2-coupled, lipid raft-localized EAAT1 underlies perturbed circadian regulation in glia-specific *cpes* knock-downs. (A) and (B) Knock-down of *EAAT1* (A) and *Nrv2* (B) in glial cells perturbed locomotor rhythm. Circadian power and period were decreased in both *Repo > EAAT1-IR* (*n* = 6) and *Repo > Nrv2-IR* (*n* = 7) compared to control flies *Repo > white-IR* (*n* = 17). *P*-values from Student’s *t*-test are indicated. (C) Schematic model of aberrant transynaptic glutamate signaling under glial-specific *cpes* knock-down compared to control conditions. Reduction in glia CPE disrupts the activity of glia-resident EAAT1 localized in CPE-enriched raft microdomains. The corresponding activity of the Na^+^/K^+^-dependent ATPase pump (Nrv2), which is coupled to EAAT1 glutamate intake, decreases. Reduction in ATP consumption decreases glucose uptake and lactate production in glia cells, cutting the neuronal energy supply. In addition, impeded glutamate uptake by glia cells triggered compensatory metabolic conversions of glutamate to GSH and glutamine to cope with glutamate excess. Enhanced glutamate uptake in postsynaptic neuronal induces elevated production and release of purines, e.g. adenosines, which may serve to inhibit further presynaptic glutamate release. Compromised transynaptic glutamate signaling leads to perturbed membrane potential, abated oxidative phosphorylation via the mitochondrial electron transport chain and redox disturbances. The glia-coupled postsynaptic neuron becomes incapable of propagating incoming action potentials to maintain circadian regulation.

We next conducted an extensive genetic screening to test our postulation by generating (i) a series of genetic knock-downs corresponding to individual DEPs uncovered from proteomics analysis and other relevant genes based on a literature search and (ii) rescue assays that knock-down or overexpress genes corresponding to individual DEPs under a *cpes^1^* mutant background (Supplementary Dataset 2). A total of 540 genetic screens were created and examined for perturbations in locomotor rhythm, of which 70 strains were found to display altered circadian rhythm. Corroborating our preceding results, a list of genetic knock-downs implicating transynaptic glutamate signaling and clearance was identified from the screen, which include genes coding for the glutamate receptors, e.g. *VGlut, GluRIIA, GluRIIB, nmdar1* and *nmdar2*, the glial-resident glutamate transporter *Eaat1* and the Na^+^/K^+^-ATPase pump *nrv2* and *nrv3*, to which the activity of EAAT1 is coupled (Supplementary Dataset 2). The actograms of *Repo > Eaat1-RNAi* and *Repo > Nrv2-RNAi* were altered compared to control flies and both circadian power and period were significantly reduced compared to *Repo > white-IR* controls (Fig. 7A and B). In contrast, the circadian rhythm of *Repo > Eaat2-RNAi*, which does not transport glutamate but preferentially takes in aspartate and taurine (Besson *et al.*, 2005), was not significantly changed (Supplementary Fig. S8E). These observations showed that the dysregulation in transynaptic regulation underlying arrhythmic locomotor activity in *Drosophila* is predominantly attributed to aberrant glutamate signaling and altered glutamate expenditure.

All of the rescue assays corresponding to DEPs uncovered from proteomics, however, did not rescue circadian rhythm under a *cpes^1^* mutant background. Only three rescue screens that express the *Drosophila* CPES (*cpes^1^; Repo > CPES*) and human sphingomyelin synthases SGMS1 and SGMS2 (*cpes^1^; Repo > SGMS1, cpes^1^; Repo > SGMS2*) exhibited partial rescue, while the *nSMase^1^; cpes^1^* double mutation showed a total rescue (*cpes^1^; nSMase1*) (Supplementary Dataset 2). These observations imply that reductions in CPE per se disrupt circadian rhythm via aberrant neural–glial glutamate signaling that is less dependent on the actual abundances of individual metabotropic and ionotropic receptors involved, but more on the endogenous abundance of CPE instead.

## DISCUSSION

Astrocytes modulate the extracellular milieu between presynaptic and postsynaptic neuronal membranes via clearance of neurotransmitters and/or release of signaling metabolites to elicit synaptic activity, which together constitute the tripartite synapse [63]. Synchronous astrocytes are able to entrain rhythmicity in neurons either by releasing synaptically active molecules or via the provision of metabolic substrates (in the form of lactate) to fuel neuronal activity [64]. Previous works using electron microscopy demonstrated that ALG are located in the proximity of insect neuropils, in a manner akin to mammalian astrocytes, and they express EAATs to salvage excess synaptic glutamate (or other metabolites such as aspartate) in preparing the local synaptic environment for incoming synaptic transmission [65].

In mammalian systems, SM (functional analogs of CPE), alongside cholesterol and glycosphingolipid, form specialized membrane microdomains of higher viscosity termed lipid rafts, also commonly referred to as ‘detergent-resistant membranes’ (DRMs). The clustering of ionotropic/metabotropic neurotransmitter receptors and transporters in lipid rafts critically alters the potency and efficacy of neurotransmission [66]. The mammalian homolog of the glial-resident glutamate transporter (EAAT2), which shares 35% amino acid identity with *Drosophila* EAAT1 [54], is concentrated in lipid raft microdomains [67,68]. Lipid raft localization of mammalian EAAT2 is crucial to the Na^+^-dependent glutamate uptake by glial cells, which underscores the importance of the membrane lipid landscape in mediating synaptic glutamate metabolism. In *Drosophila*, CPE denotes the major constituent that determines the formation of DRMs. Indeed, a lack of CPE greatly compromises DRM formation in *cpes* mutants and the detergent-resistant membrane band was restored when *cpes* mutants were rescued with *UAS-CPES* [31].

Our present findings demonstrate that the endogenous level of CPE in the plasma membrane of ALG is crucial to the maintenance of circadian rhythm in *Drosophila*. CPE reductions in *cpes* knock-downs and *cpes^1^* mutants were accompanied by arrhythmic locomotor activity, while both pan-glial-specific and ubiquitous elevations of CPE in overexpression lines largely restored locomotor activity. Our findings revealed the essential role of CPE in maintaining circadian rhythm that is modulated by ALG. In line with our findings, numerous preceding studies have demonstrated the role of neural–glia coupling in the regulation of circadian rhythm in *Drosophila*. For example, perineurial glia that forms the blood–brain barrier was found to be a predominant source of nitric oxide that fine-tunes circadian locomotor output. Perineurial glia-specific knock-down of nitric oxide synthase resulted in behavioral arrhythmia partly attributable to malformed neurites of the main pacemaker neurons [69]. Glial expression of Ebony (*N*-β-alanyl-biogenic amine synthase) rescues perturbed circadian behavior in *ebony* mutants, likely mediated by glia coupling with dopaminergic neurons. Normal neuronal output of clock genes in *ebony* mutants indicated a role for Ebony downstream of clock genes [70]. In *cpes* knock-downs, however, molecular oscillations in clock genes were altered. Precise mechanistic links connecting how endogenous CPE levels modulate clock genes expression are not elucidated herein, but we think that CPE-enriched lipid rafts may be involved. In a similar light, selective serotonin reuptake inhibitors were reported to alter the rhythmic expression of *per* in rat-1 fibroblasts in a raft-dependent manner [71]. The activities of sphingolipid-metabolizing enzymes, including SMS2 and nSMase, regulate the secretion of exosomes that mediate neural–glia communication [72] and exosome trafficking has been implicated in the control of clock gene expressions that is mediated by exosome microRNAs cargoes in mice [73].

Comparative proteomics between control and *cpes* glial knock-down flies revealed that synaptic glutamate utilization and transport, mediated by EAAT1, are dysregulated in CPE-deficient flies, accompanied by compromised mitochondrial electron transport chain function and increased oxidative stress that denote hallmark features of glutamate excitotoxicity. In line with this, it was previously reported that downregulation of EAAT1 in fly brains resulted in elevated oxidative stress and mitochondrial swelling, which make the flies less active with increased sensitivity to paraquat, illustrating a reduced capacity to cope with oxidative stress. EAAT1-deficient flies also exhibited signs of neurodegeneration, marked by a modest loss of dopaminergic neurons, and possessed a shortened lifespan, which were attributed to decreased glutamate-buffering capacity [55]. Lifespan reduction and diminished antioxidant capacity were also observed in *cpes^1^* mutants and glial-specific *cpes* knock-down flies in our study, showing that these might be common downstream behavioral/metabolic traits orchestrated by dysregulated synaptic glutamate homeostasis, either via reduction of EAAT1 protein or, in our case, a loss of lipid raft membrane microdomains crucial to EAAT1 function.

Interestingly, it was observed that the embryos and larvae of EAAT1 knock-down flies did not present any signs of neurodegeneration and behavior deficits, which the authors attributed to a possibly reduced sensitivity of *Drosophila* to glutamate toxicity in earlier developmental stages [55]. In contrast, we found in our study that membrane CPE depletion underlying arrhythmic locomotor activity is detrimental even at developmental stages before eclosion. This suggests that a perturbed membrane lipid landscape deficient in raft microdomains elicits a deeper and far-reaching impact on glial–neural communication compared to the loss of a single glutamate transporter itself. Indeed, it is likely that the activities of an array of neural membrane receptors and transporters were negatively impacted by the loss of lipid rafts resulting from deficient membrane CPE, as evident in the GO-MF analysis that revealed suppressed transmembrane transporter activity in *cpes* knock-downs (Fig. 6D). This also explains, at least in part, why our rescue attempts via knocking in a single gene failed to rescue the arrhythmic locomotor behavior under a *cpes^1^* mutant background. Only expression of the human SGMS1/SGMS2 that catalyses the biosynthesis of SM, the functional analogs of CPE, or direction expression of CPES rendered partial but significant rescue in *cpes^1^* mutants. While it is possible that endogenous CPE reductions lead to abnormal glia development that may subsequently preclude normal neuronal firing and circadian regulation, the relevance of membrane CPE in regulating synaptic glutamate levels and altered locomotor rhythm should not be simply overlooked. In *Drosophila*, rhythmic behavior can be mediated through controlling the pace of development rather than in the form of an acute on/off switch, as illustrated in the prototypical example on the circadian control of adult emergence (eclosion) by regulating the timing of metamorphosis completion [74]. Furthermore, it is also possible that in the absence of CPE, perturbed membrane organization impedes the formation of critical biophysical assemblies that preclude the normal functioning of membrane receptors and transporters during development. Hence, the effects of circadian regulation and developmental control cannot be easily disentangled. In addition, we observed that glia-specific *cpes* overexpression in aged flies induced at 35 days post-eclosion enhanced circadian power and greatly extended lifespan, indicating that glia-localized CPE exerts positive effects on circadian regulation and lifespan even at post-development.

Mouse astrocytes possess autonomous Ca^2+^ oscillation and entrain clock outputs [75]; the knock-down of clock genes in ALG alone, however, did not elicit arrhythmic locomotor activity in *Drosophila* (Supplementary Dataset 2). This showed that ALG regulation of the circadian rhythm is coupled to neuronal activity in *Drosophila*. We postulate that when the endogenous level of membrane CPE decreases, compromised membrane lipid rafts hinder the proper functioning of raft-localized EAAT1 in ALG, which (i) decreases the activity of the Na^+^/K^+^ ATPase pump coupled to EAAT1 and (ii) results in an accumulation of synaptic glutamate that impedes glutamate recycling to the presynaptic neuron via the glutamine–glutamate cycle. These glia-elicited changes affect neuronal activity in a dual manner. Metabolically, a reduction in ATP utilization by the Na^+^/K^+^ ATPase pump decreases glucose uptake and lactate production in glia that may compromise the primary source of metabolic fuel to neurons. Indeed, cerebral lactate was substantially reduced under *cpes* loss of function. In addition, perturbed glutamate reuptake into ALG may trigger glutamate excitotoxicity and dysregulated membrane potentials. While metabolomics analysis at the level of the whole brain did not reveal an overall elevation of cerebral glutamate, proteomics and metabolomics analyses point to compensatory metabolic conversions of glutamate to GSH and glutamine to cope with glutamate excess. These metabolic conversions that expend excess glutamate, coupled with localized increases in purines and GBH that modulate further presynaptic glutamate release and inhibit postsynaptic transmission, respectively, serve to protect against massive neuronal death under excitotoxicity (Fig. 7C). These may explain the absence of appreciable changes in the gross distribution of neurons, despite ALG being heavily compromised under *cpes* loss of function. Nonetheless, the coupled neurons are metabolically compromised and unable to propagate incoming membrane potentials effectively, leading to disturbances in locomotor activity and disruption in circadian rhythm. Along this line, Nrv2 had been implicated in human neurodegenerative disorders and aberrant Nrv2 function alters the learning and motor activity of mice [76,77]. Endogenous mRNA and protein levels of Nrv2 exhibit daily rhythmic oscillations that are absent in clock arrhythmic *Drosophila* mutants [78,79]. In our genetic screen, we also found that knock-down of *Nrv2* in glia leads to arrhythmic locomotor behavior, demonstrating that the activity of the Na^+^/K^+^ pumps itself is also critical in regulating metal-ion concentrations and membrane potentials key to normal circadian behavior.

### Limitations of study

This study has limitations. While we identified several DEPs associated with the disruption of circadian rhythm in *Drosophila*, this study did not establish causal relationships between the identified DEPs and circadian regulation. Rescue of normal locomotor activity brought about by overexpression of the corresponding DEPs under a *cpes^1^* mutant background is imperative for the establishment of causal effects. In addition, our proteomics investigation to elucidate DEPs associated with circadian disruption under a *cpes^1^* mutant background was based only upon one single time point. An ideal set-up should comprise comparative proteomic analyses across multiple time points within the 12-h light/12-h dark cycle. Our heterologous expressions of human SGMS1 and SGMS2 in *cpes^1^* mutants showed that glia-dependent regulation of circadian rhythm in *Drosophila* requires the catalytically active production of CPE. We did not, however, directly work on flies transgenic for a catalytically inactive form of CPES, which could be produced via substitution of one of the last two aspartate residues in the high-conserved CAPT motif [D(X2)DG(X2)AR(X7-12)G(X3)D(X3)D] of CPES [30,80,81]. This study also did not examine whether defective wrapping of peripheral axons may contribute to arrhythmic locomotor activity in *cpes* mutants, given a reported role of CPE in modulating axon ensheathment in the peripheral nervous system of *Drosophila* [82]. The morphology of individual ALG cells and their relationship to neighboring neurons under *cpes* loss of function was not elucidated herein, which could be achieved by generating mosaic flies using the multicolor flip-out technique [44,83]. Finally, we did not directly measure glutamate levels in synaptic clefts using the glutamate sensor iGluSnFr, which is anchored to the exterior of the plasma membrane and senses extracellular glutamate within the synaptic clefts [84].

In summary, we report herein that membrane CPE plays an essential role in modulating circadian behavior in *Drosophila*, mediated principally via neural–glia coupling in the regulation of synaptic glutamate homeostasis. Our findings also suggest that region-specific elevations of functional membrane lipids can benefit circadian regulation, even at post-development stages.

## MATERIALS AND METHODS

Further information on materials used to conduct the research and methodological details used in the analyses are available in the Supplementary data.

### Circadian behavior Analysis

Spontaneous locomotor activity was recorded using a pySolo video monitoring system and data analysis was performed using pySolo program files and Matlab software, as described previously [85]. Free-running activity under constant darkness was analysed using a Clocklab (Actimetrics) to calculate the period and power. Flies were raised at 25^o^C, 60% relative humidity, ∼800-lux LED white light, 12-h light/12-h dark conditions. All experiments to monitor locomotor activity were conducted using flies on Days 5–7 after eclosion.

### Omics analyses

Lipidomic analyses were performed as described [86,87] and data were expressed in μmoles per fly/per head. A full proteomics data set is provided in Supplementary Dataset S3. Untargeted metabolomics was conducted using an Agilent 1290 II UPLC coupled to a Sciex 5600 + Triple TOF [88] and the metabolomics data set is provided in Supplementary Dataset S4.

### Immunohistochemistry and microscopy

Fly brains from *Repo:: mCD8-gfp* and *Alrm:: ChRFP* strains were dissected in cold phosphate-buffered saline, then fixed with 4% formaldehyde in PBST (containing 0.3% Triton X-100) for 45 mins at 25°C. Brains were washed for three rounds using PBST, 15 mins per round on a shaker. Brain samples were blocked using 5% goat serum for 30 mins, mounted using VECTASHIELD Mounting Medium containing DAPI and imaged using a Leica SP5 II confocal microscope at 1-μm intervals. A Z stack model was taken to image the 3D view and pictures were processed using Image J. Flies without linked fluorescent protein were incubated with rat anti-Elav (DSHB, 7E8A-10, 1:100) primary antibody diluted in PBST after blocking for 12 h, washed three times using PBST (as described above) and then incubated with goat anti-Rat IgG Alexa Fluor 555 secondary antibody (1:200) overnight at 4°C. Samples were washed three times using PBST and then mounted and imaged as aforementioned and according to a previous study [52].

### Statistical analysis

Data were analysed using GraphPad prism 8.0; error bars in the figures represent SEM. Fisher's exact test was used for analysis of circadian-related data. For analysis of proteomics data, all UniProt IDs from all protein groups were mapped to entrez IDs via maplds function using the *Drosophila melanogaster* database (org.Dm.eg.db 3.10.0). Only the first entrez ID was obtained corresponding to each UniProt ID. Protein groups that mapped to only one entrez ID were retained for GSEA. The gene list is ranked using t-statistics. GSEA was performed using gseGO function from R package clusterProfiler 3.14.3 with a *P*-value cut-off set at <0.05. Other statistical details are described in the Supplementary data.

## Supplementary Material

nwac148_Supplemental_Files
